# 7^th ^SOSORT consensus paper: conservative treatment of idiopathic & Scheuermann's kyphosis

**DOI:** 10.1186/1748-7161-5-9

**Published:** 2010-05-30

**Authors:** JC de Mauroy, HR Weiss, AG Aulisa, L Aulisa, JI Brox, J Durmala, C Fusco, TB Grivas, J Hermus, T Kotwicki, G Le Blay, A Lebel, L Marcotte, S Negrini, L Neuhaus, T Neuhaus, P Pizzetti, L Revzina, B Torres, PJM Van Loon, E Vasiliadis, M Villagrasa, M Werkman, M Wernicka, MS Wong, F Zaina

**Affiliations:** 1 Clinique du Parc, 155 bd Stalingrad, 69006 Lyon, France; 2 Orthopaedics and Traumatology Department Bambino Gesù Children's Hospital, 4, P. S. Onofrio, Roma, RM 00165, Italy; 3 Orthopaedic Department, Rikshospitalet University Hospital, NO-0027 Oslo, Norway; 4 Department of Rehabilitation University Hospital Medical Center of Silesia, Warszawska 14 40-006 Katowice, Poland; 5 ISICO (Italian Scientific Spine Institute), Via R Bellarmino 13/1, 20141 Milan, Italy; 6 Orthopaedic Department, "Thriasio" General Hospital, G. Gennimata, Av. 19600, Magoula, Attica, Greece; 7 Department of Orthopaedic Surgery, Research School Caphri, PO Box 616,6200 MD, Maastricht University Medical Centre, the Netherlands; 8 Department of Pediatric Orthopedics and Traumatology University of Medical Sciences, 10 Fredry Street 61-701, Poznan, Poland; 9 Centre medico-chirurgical et de réadaptation des Massues, 92, rue Edmond-Locard, 69322 Lyon cedex 05, France; 10 474 Lansdowne Rd. N, Ottawa Ontario K1M0X9, Canada; 11 Posturetek 2823 Boulevard Rosemont Montréal, Canada; 12 8 Mivtza Kadesh St., 71720 Modi'in, Israel; 13 ha'chartzit 10th, rishon le-zion, Israel; 14 3380 St. Michael Dr., Palo Alto, CA 94306, USA; 15 Slingeland Ziekenhuis Kruisbergseweg 25 7009 BL Doetinchem, The Netherlands; 16 Institut E. Salvá, Vía Augusta 185, 08021, Barcelona, Spain; 17 Orthopedic Rehabilitation Services, Alzeyer Str. 23, D-55457 Gensingen, Germany; 18 SCOLIOCARE-ORTHOMED, Gesundheitsforum Nahetal, Alzeyer Strasse 23, 55457 Gensingen Germany; 19 University School of Physical Education, Department of Kinesitherapy, Królowej Jadwigi 27/39, 61-871, Poznan, Poland; 20 IDepartment of Health Technology and Informatics, The Hong Kong Polytechnic University, Hong Kong Polytechnic University, 11 Yuk Choi Rd, Hung Hom, Hong Kong, China

## Abstract

Thoracic hyperkyphosis is a frequent problem and can impact greatly on patient's quality of life during adolescence. This condition can be idiopathic or secondary to Scheuermann disease, a disease disturbing vertebral growth. To date, there is no sound scientific data available on the management of this condition. Some studies discuss the effects of bracing, however no guidelines, protocols or indication's of treatment for this condition were found. The aim of this paper was to develop and verify the consensus on managing thoracic hyperkyphosis patients treated with braces and/or physiotherapy.

**Methods:**

The Delphi process was utilised in four steps gradually modified according to the results of a set of recommendations: we involved the SOSORT Board twice, then all SOSORT members twice, with a Pre-Meeting Questionnaire (PMQ), and during a Consensus Session at the SOSORT Lyon Meeting with a Meeting Questionnaire (MQ).

**Results:**

There was an unanimous agreement on the general efficacy of bracing and physiotherapy for this condition. Most experts suggested the use of 4-5 point bracing systems, however there was some controversy with regards to physiotherapeutic aims and modalities.

**Conclusion:**

The SOSORT panel of experts suggest the use of rigid braces and physiotherapy to correct thoracic hyperkyphosis during adolescence. The evaluation of specific braces and physiotherapy techniques has been recommended.

## Background

Kyphosis can be paradoxically more difficult to treat than scoliosis. There are many types of kyphosis that require various strategies of treatment. Furthermore, there is only a little evidence on the conservative treatment of kyphosis, less than that on scoliosis. With lacking information on the natural history, the difficulties of clinical and radiological assessment, the unclear definition of normal kyphosis and the variety of clinical forms and etiology, vague indications of treatment are allowed for at best. Untreated, kyphosis in the growing child may lead to a progressive deformity of the spine and back pain. At birth, the entire spine shows a slight posterior curve from the occiput to the sacrum. When the baby begins to hold his head up, a cervical lordotic curve develops. However with the sitting position a total kyphosis can be encouraged. With weight bearing and ambulation, the pelvis tilts forward and a lumbar lordosis develops. Staffel [1] classified human posture into three distinct groups: "round"," flat" and "lordotic."

In the early twentieth century, radiographs allowed Scheuermann [2] in Copenhagen to describe and illustrate anterior wedging of vertebral body.

In 1939, Schmorl [3] described the alterations of the growth plate of the vertebral body and nodules that bear his name.

In 1964, in his monograph Sorenson [4] does not find a solution to correct kyphosis.

Subsequently many authors have proposed normal ranges of posterior sagittal thoracic curves of: 20°-40° for Roaf [5] and 30 ° for Rocher [6].

The great variance in the range of curve angles in thoracic kyphosis may rely on the radiological position it was taken. In 1982, Stagnara de Mauroy et al. [7] defined a radiological position similar to that of a clinical examination and using a computer calculated the reciprocal angle of vertebral bodies in the sagittal plane.

The pathogenesis of Scheuermann's disease was described in 1986 by Aufdermaur [8]. It is a disorders of endochondral ossification of the vertebral bodies. The marginal border (vertebral rim) is intact, allowing the reconstruction of the anterior wall with conservative orthopedic treatment.

Ippolito [9] highlights the structural abnormalities of cartilage being very thin with collagen fibrils and irregularity of mineralization and ossification of the vertebral plates.

In 1977, White and Panjabi, [10] describe the biomechanics of kyphotic deformities in the sagittal plane and justify treatment by bracing. Kyphosis develops when the balance between the load bearing capacity of the anterior and posterior elements of the spine are disrupted. Kyphotic deformities can be treated with bracing that reduce the axial load and shift the center the gravity.

in 1999, Wenger and Frick [11] published an extensive review on this condition, but when looking into recent Pub Med listings, the condition of Scheuermann's kyphosis in the past 10 years seems to stimulate less scientific interest. There are some points of discrepency upon the definition of the pathological deviations of normal and sagittal spinal alignment [11,12]. Unlike scoliosis, where any significant lateral deviation in the coronal plane is abnormal, the sagittal alignment of the spine has a normal range of thoracic kyphosis. The Scoliosis Research Society has defined this range as being from 20° to 40° in the growing adolescent [13-15]. In a study of 316 healthy subjects with ages ranging from 2 to 27 years, the upper limit of normal kyphosis was noted to be 45°. It was also noted that the average thoracic kyphosis increases with age from 20° in childhood, to 25° in adolescents, to 40° in adults [16]. The lack of a consistent definition of Scheuermann's kyphosis in the literature makes it difficult to compare studies as the inclusion criteria may differ, thus making the distinction between the spectrum of upper normal thoracic kyphosis, severe adolescent roundback deformity, and Scheuermann's disease almost impossible [11,12].

Little is written on the subject of the lumbar or thoracolumbar patterns of Scheuermann's disease. The Schmorl's nodes and endplate irregularity may be so severe that the lower lumbar Scheuermann's disease can be confused with infection, tumor, or other conditions [11]. The etiology of lumbar Scheuermann's kyphosis is unknown, but strong associations with repetitive activities involving axial loading of the immature spine favour a mechanical cause [11]. Although the radiographic appearance may be similar, lumbar Scheuermann's kyphosis is regarded as a different entity than thoracic Scheuermann's kyphosis [11]. Unlike classic thoracic Scheuermann kyphosis, the treatment of lumbar Scheuermann's disease was not controversial in 1999 [11], as its course has been regarded as being non-progressive and its symptoms have been regarded to resolve with rest, activity modification and time [17,18].

The loss of lordosis in this area of the lumbar or thoracolumbar spine means that Scheuermann's disease can be one of the predictors of developing chronic low back pain in adulthood:

Loss of lumbar lordosis correlates well with the incidence of chronic low back pain in adulthood [19,20]. Sedentary lifestyles contributes to a loss of lumbar lordosis as well as scoliosis and thoracolumbar or lumbar kyphosis [21]. It is necessary to recognise that the severity of symptoms in patients with back pain increase in a linear fashion with progressive sagittal imbalance. The results of these studies also show that hyperkyphosis is more favourable in the upper thoracic region but very poorly tolerated in the lumbar spine [19-21]. As it has been shown that restoring lumbar lordosis stabilises the spine with respect to lateral deformity [22], so we may assume that lumbar decreased lumbar lordosis or lumbar kyphosis destabilises the spine and can lead to chronic low back pain [23,24]. Ten years after the review by Wenger and Frick [11], lumbar Scheuermann's disease should have been investigated specifically, focussing upon the prevention of chronic low back pain in adulthood [12].

This discussion has been opened after the time this consensus paper has been planned, and it has been presented at the SOSORT Lyon meeting at the time the Delphi process was already ongoing. Therefore not all of the latest updates have been included into the questionnaires. Nevertheless the consensus papers are part of a constant process of development and therefore this actual discussion will surely be part of future consensus papers on this topic.

The conservative orthopedic treatment with casts and braces was developed in parallel to that of scoliosis 60 years ago. This treatment is intended primarily for normal idiopathic kyphosis or kyphosis developing secondary to Scheuermann's disease. Wearing a brace can prevent the collapse of the anterior wall of the vertebral body and in some cases of Scheuermann's, to help reform this.

Due to this lack of understanding a Consensus among experts can at this point give some more understandings about this neglected area of research. Since 2005, SOSORT is developing systematically and yearly Consensuses on the different areas of conservative treatment of spinal deformities [25-30]. The aim of this paper is to report on the last SOSORT Consensus focused on "Conservative Treatment of Idiopathic & Scheuermann's Kyphosis".

## Methods

The Delphi method was used which is a systematic, interactive forecasting method which relies on a panel of experts. The experts answer questionnaires in two or more rounds. After each round, a facilitator provides an anonymous summary of the experts' forecasts from the previous round as well as the reasons they provided their comments. Thus, experts are encouraged to revise or clarify their earlier answers in light of the replies of other members of their panel. It is believed that during this process the range of answers will decrease and the group will converge towards the "correct" answer. Finally, the process is stopped after a pre-defined stop criterion (Q26 & 27) and the mean or median scores of the final rounds determine the results[31].

Each question (Q) is deemed positive or negative by Consensus. Due to the low Consensus in general on this topic we split the possible Consensus as follows: Strong Consensus (over 90%), Good Consensus (75%-89%), Weak Consensus (51-74%)

As some questions were added in the second and third round. the order of questions were modified to make the text easier to read.

## The primary questions

*Q1 Title: Bracing and physiotherapy can be useful for Juvenile Kyphosis and can modify the natural progression*.

*Results*: For the **Q1 **we have **100% **positive answers. This point has achieved Consensus.

*Comment*: Restoration of lordosis at the Thoracolumbar area is proven effective.

*Discussion: *In literature [32] the answer is different and many authors believe that conservative treatment is inneffective and not justified in view of tolerance to adulthood. In this consensus we have grouped experts in the conservative treatment.

Q2 Title: Do you treat Kyphosis conservatively at your Center?

*Results*: For the **Q2 **we have also **100% **of positive answers. This point has achieved Consensus.

*Discussion: *This confirms the previous question.

## Physiotherapy

*Q3 & 4 Title*: *What are the therapeutic aims of physical exercise in the treatment of patients at risk of brace and what is the priority?*

*Results*: We have given a score according to priority: 3 = high priority, 2 = medium priority, 1 = low priority, 0 = non selected. The results are presented in order of importance of the score.

**Figure 1:**
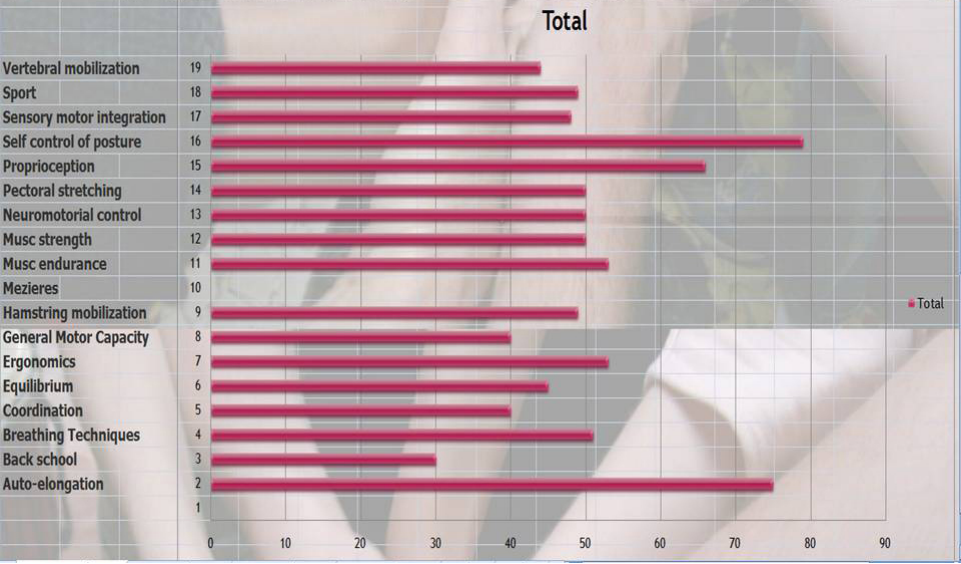
**Therapeutic aims of physical exercise treatment in patients at risk of brace**.

Self control of posture (79), auto-elongation (75), Proprioception (66), Muscular endurance (53), ergonomics (53), Breathing techniques (51), Pectoral stretching (50), Neuromotorial control (50), Muscular strengthening (50), Sport (49), Hamstring mobilization (49), Sensori-motor integration (48), Equilibrium (45), Vertebral mobilization (44), General Motor capacities (40), Coordination (40), Back school (30). [See figure 1]

*Comment*: The purpose is to correct them in auto-elongation looking for the best 3D correction (mainly based in the sagittal profile) and stabilize it isometrically. Then train them to automatically change to this new posture.

*Discussion: *Looking at Q3 and Q4, it appears that self control posture {79/81 = **98%**} and auto-elongation {75/81 = **93%**} with the use of proprioception are the most used techniques. The back school is rarely used. It is difficult to see a consensus on other physiotherapy techniques, which reflects the diversity of kyphosis.

Q15 Title: Do you usually give some "home exercises" to practice daily? If yes, how long every day.?

*Results: *Yes (8), No (3), 10 minutes (1), twenty minutes (4), thirty minutes (2), more (2)

[See figure 2]

**Figure 2:**
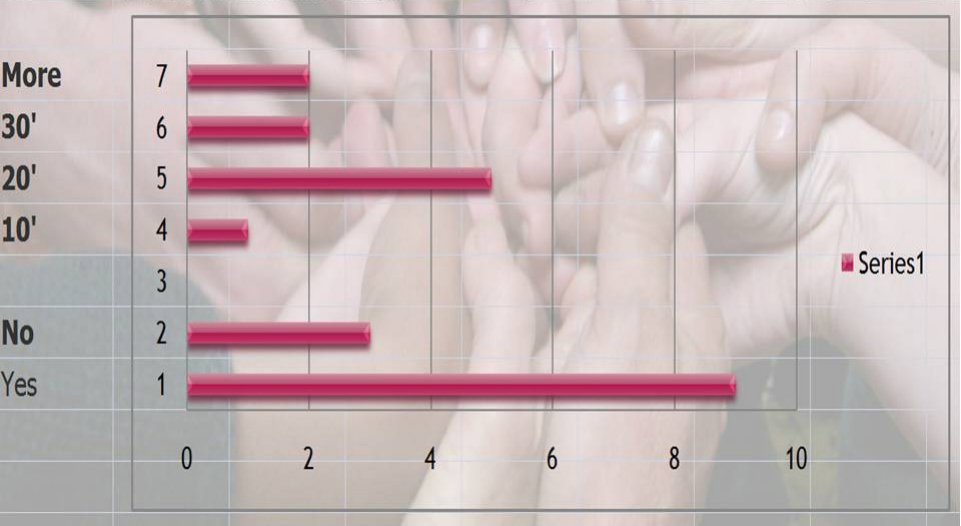
Do you usually give some "home work" to practice daily? If yes, how long every day?

*Comments*: Advised to avoid too much sitting and change their sitting position often. Use the prone position for reading and watching TV. Sit on the edge of a chair with alternative extension on one hip. I encourage them to do more (the important thing is not just the time, it is the way they are doing it) the level of correction and concentration while they do it because this will be more successful for them.

*Discussion: *This question was added after the comments from the first round. We have a consensus for home exercises and the average time is 20 minutes, which in France is the official duration of a session of physiotherapy exercises in vertebral deviation.

## Indications

Q5 & 6 Title: Why do we treat Kyphosis and the priority of this?

*Results*: Like Q 3 & 4 we have given a score according to priority.

Scheuermann (74/81 = **91%**), Back pain (72/81 = **89%**), Quality of life (69/81 = **85%**), Aesthetics (67/81 = **83%**), Progression in adulthood (58/81 = **72%**), Psychological well being (57/81 = **70%**), Cobb degree (55/81 = **68%**), Disability (43/81 = **53%**), Breathing Function (38/81 = **47%**).

[See figure 3]

**Figure 3:**
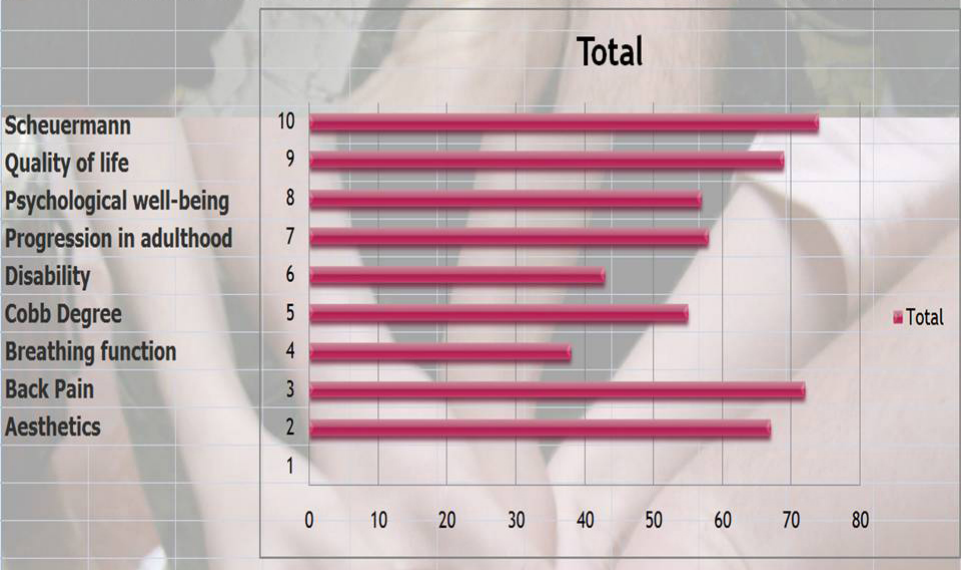
Why do you treat kyphosis?

*Discussion*: There is a consensus for Scheuermann and back pain which is often the same. We also have a consensus on breathing function. Indeed there is no relationship between the angle of kyphosis and vital capacity.

Q7 & 8 Title: What information do you need before treatment and its priority?

*Results*: As with previous questions, we assigned a score for each item.

**Figure 4:**
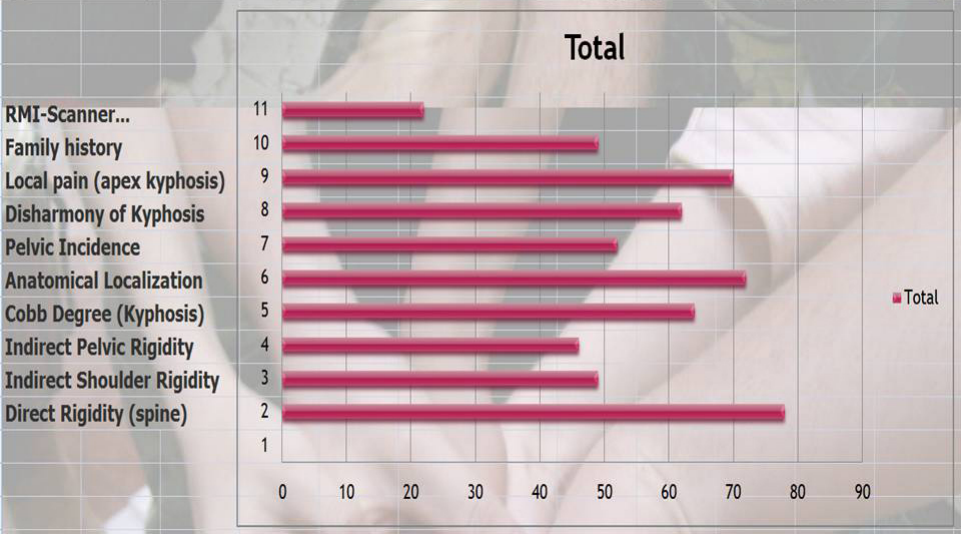
Which information do you need before treatment?

Direct rigidity of the spine (78/81 = **96%**), Anatomical localization (72/81 = **89%**), Local pain at the apex of kyphosis (70/81 = **86%**), Cobb degree (64/81 = **79%**), Disharmony of kyphosis (62/81 = **77%**), Family history (49/81 = **60%**), Indirect shoulder rigidity (49/81 = **60%**), Indirect pelvic rigidity (42/81 = **52%**), RMI-scanner (22/81 = **27%**) [See figure 4]

*Discussion: *We have a consensus on the rigidity of the curve, anatomical location and local pain. Unlike scoliosis, the Cobb angle is less important. There is also consensus for not requiring further radiological examinations like RMI and scans.

## Bracing

Q9 & 10 Title: With regards to bracing: What is your management and how do you prioritize it?

**Figure 5:**
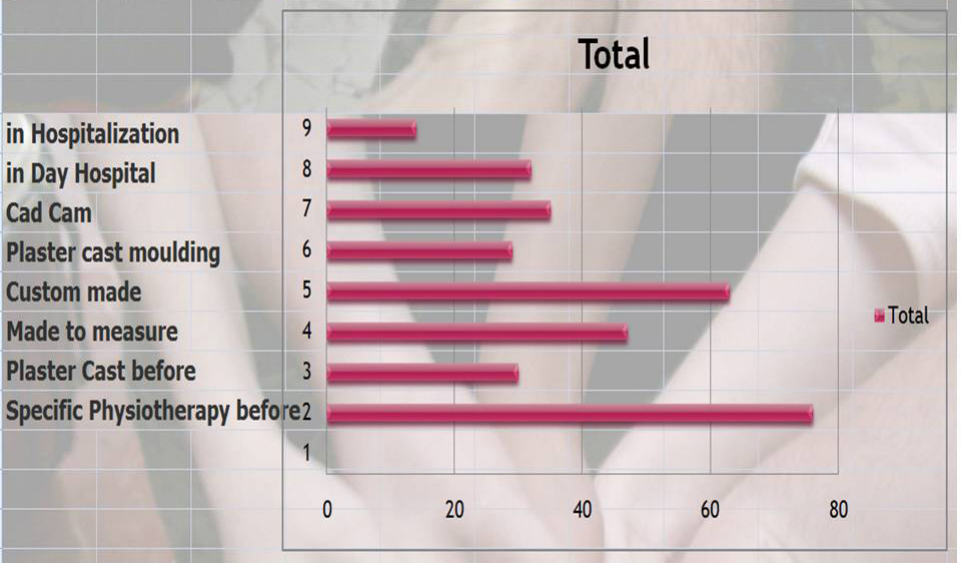
Finally bracing: what is your management?

*Results: *Specific physiotherapy before (70/81 = **86%**), Custom made (63/81 = **78%**), Made to measure (47/81 = **58%**), Cad Cam (35/81 = **43%**), In day Hospital (32/81 = **39%**), Plaster cast before (30/81 = **37%**), Plaster cast moulding (29/81 = **36%**), in Hospitalization (14/31 = **17%**) [See figure 5]

*Discussion: *We have achieved a consensus for physiotherapy before bracing and no hospitalization {67/81 = **83%**}.

Q16 Title: What are the physiological reasons for the patient to wear the brace and its priority?

**Figure 6:**
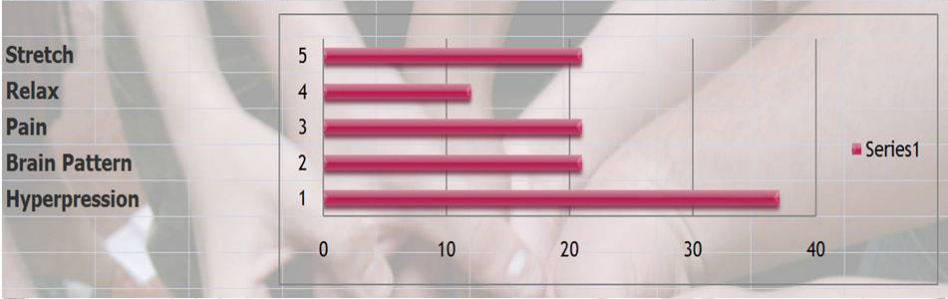
What are the physiological reasons for the patient to wear the brace?

*Results: *To avoid hyperflexion on the anterior wall (36/36 = **100%**), discourage bad posture (19/36 = **53%**), Pain (19/36 = **53%**), Stretch(19/36 = **53%**), relax (10/33 = **30%**) [See figure 6]

*Comment*: To restore proper alignment of muscular forces. To give the thoracolumbar discs space again for proper development by reducing continuous loading.

*Discussion*: We have a consensus with the biomechanical approach of White and Panjabi.

Q17 Title: According to your experience and results -- what are the main criteria for an unsuccessful treatment (physiotherapy or brace) and their priority?

**Figure 7:**
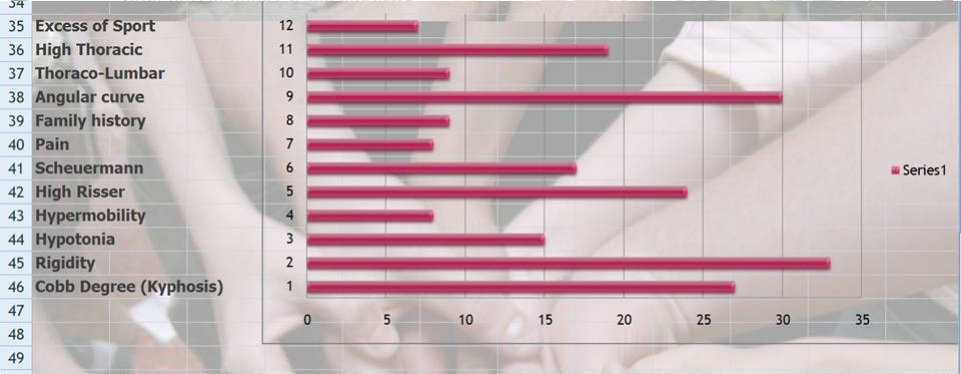
According to your experience and results -- what are the main criteria for an unsuccessful treatment (physiotherapy or brace)?

*Results: *Rigidity (33/33 = **100%**), Angular curve (30/33 = **91%**), Cobb degree (27/33 = **82%**), High Risser (24/33 = **73%**), high thoracic curve (19/33 = **58%**), Scheuermann (17/33 = **52%**), hypotonia, (15/33 = **45%**), Thoraco-lumbar pattern (9/33 = **27%**), Family history (9/33 = **27%**), Pain (8/33 = **24%**), Hypermobility (8/33 = **24%**), Excess of sport (7/33 = **21%**) [See figure 7]

*Comment*: Compliance is the main criteria. When there is a good compliance results are good regardless of curve rigidity and Cobb angle.

*Discussion*: The hierarchy of factors making the conservative treatment difficult is a valuable indicator to justify early treatment. The clinical examination should come before radiological findings.

Q18 Title: What is your most frequent protocol of wearing the brace in adolescent **thoracic **kyphosis?

**Figure 8:**
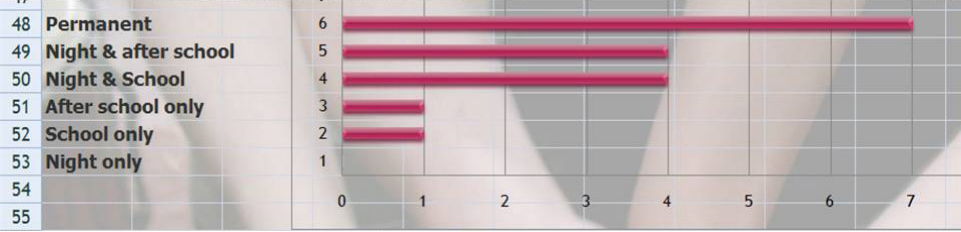
What is your most frequent protocol of wearing the brace in adolescent thoracic kyphosis?

*Results*: Permanent (7), Night & school (4) night & after school (4) after school only (1), School only (0), Night only (0) [See figure 8]

*Discussion*: The consensus for night and day wearing means that the intended effect of the brace is not only a mechanical support in the erect position but also concerns the Wolff's laws like scoliosis during nocturnal growth.

Q19 Title: What is your most frequent protocol of wearing the brace for adolescent **thoraco-lumbar **kyphosis?

**Figure 9:**
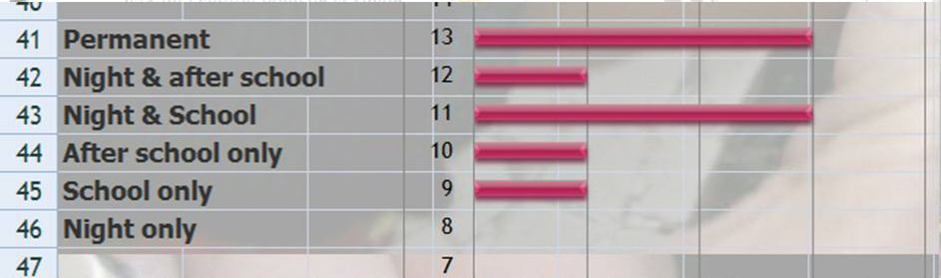
What is your most frequent protocol of wearing the brace for adolescent thoraco-lumbar kyphosis?

*Results*: Permanent (3), Night & school (3) night & after school (1) after school only (1), School only (0), Night only (0) [See figure 9]

*Discussion*: For this pattern, there is a consensus with the sitting position.

Q20 Title: What is your most frequent protocol of wearing the brace for **pre-pubertal **kyphosis?

**Figure 10:**
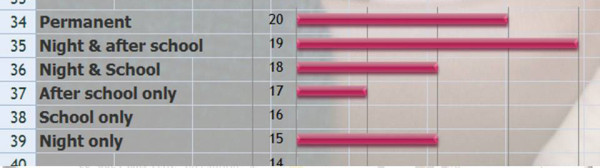
What is your most frequent protocol of wearing the brace for pre-pubertal kyphosis?

*Results*: Permanent (3), Night & school (2) night & after school (4) after school only (1), School only (0), Night only (0) [See figure 10]

*Discussion*: The majority of respondents are in favor of a part time protocol. Unlike scoliosis there is a consensus to exclude only wearing it at night which confirms the importance of factors related to postural vertical loading in kyphosis.

The following three questions were developed for the 3rd round. After the 2nd round, it became impossible to define patterns of braces. We have therefore tried to approach the consensus with the technical descriptors of these orthotics.

Q21 Title: Choose your ideal brace for adolescent **thoracic **kyphosis?

*Results: *material | plexidur (2), polypropylene (2), polyethylene low density (2), polyethylene high density (7)

Opening | lateral (5), posterior (2), anterior (4)

Level of the brace | Iliac crest (2), lateral pelvis (2), cervical (0), clavicular (3) sternal (5)

**Figure 11:**
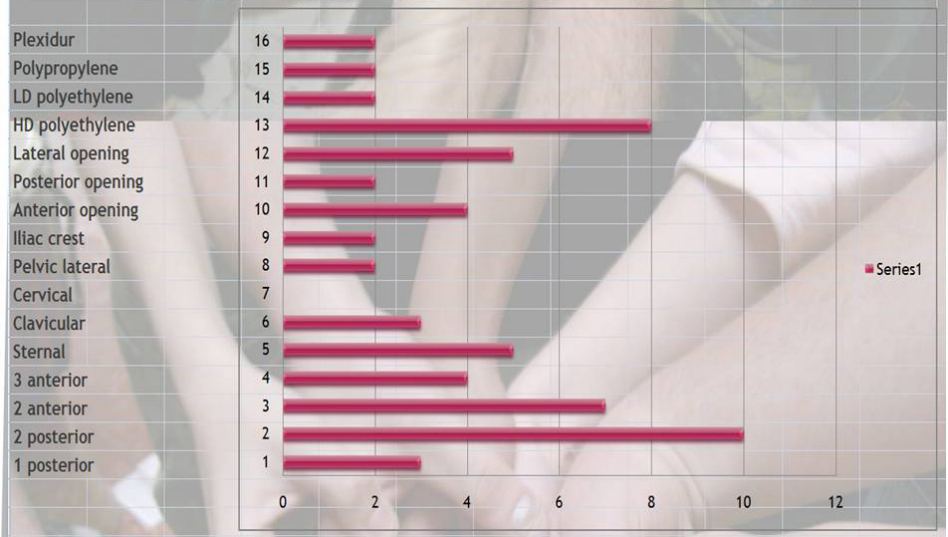
Choose your ideal brace for adolescent thoracic kyphosis?

Pressure points | 3 anterior with pelvis, inferior thorax, and sternum (3), 2 anterior point with pelvis and sternum (7), 2 posterior with sacrum and apex kyphosis (9), one posterior with apex kyphosis only (1) [See figure 11

*Discussion: *There is slight consensus for polyethylene high density material, however no consensus for brace opening and consensus for a sternal support.

The biomechanical effects can be:

3 points: one posterior, 2 anterior like traumatic kyphosis

4 points: two posterior (lordosis control) and two anterior

5 points: two posterior and three anterior for better control of lordosis.

There is a consensus for a four points system, even if it is easier to control the sagittal posture of the spine with a 5 point system. This point deserves further discussion.

Q22 Title: Choose your ideal brace for adolescent **thoraco-lumbar **kyphosis?

*Results: *material | plexidur (2), polypropylene (1), polyethylene low density (2), polyethylene high density (3)

Opening | lateral (2), posterior (2), anterior (2)

Level of the brace | iliac crest (1), pelvic lateral (2), cervical (0), clavicular (1) sternal (0)

**Figure 12:**
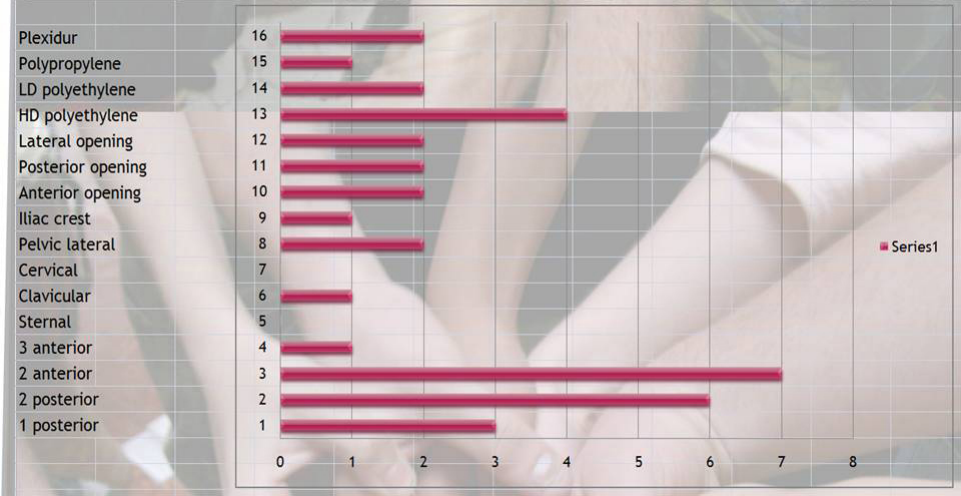
Choose your ideal brace for adolescent thoraco-lumbar kyphosis?

Pressure points | 3 anterior with pelvis, inferior thorax, and sternum (1), 2 anterior point with pelvis and sternum (6), 2 posterior with sacrum and apex kyphosis (5), one posterior with apex kyphosis only (3) [See figure 12]

*Discussion: *There is no consensus for material, no consensus for opening and consensus for a very logical 4 points system.

Q23 Title: Choose your ideal brace for **juvenile **kyphosis?

*Results: *material | plexidur (0), polypropylene (0), polyethylene low density (0), polyethylene high density (2)

Opening | lateral (0), posterior (3), anterior (0)

Level of the brace | iliac crest (0), pelvic lateral (2), cervical (1), clavicular (0) sternal (0)

**Figure 13:**
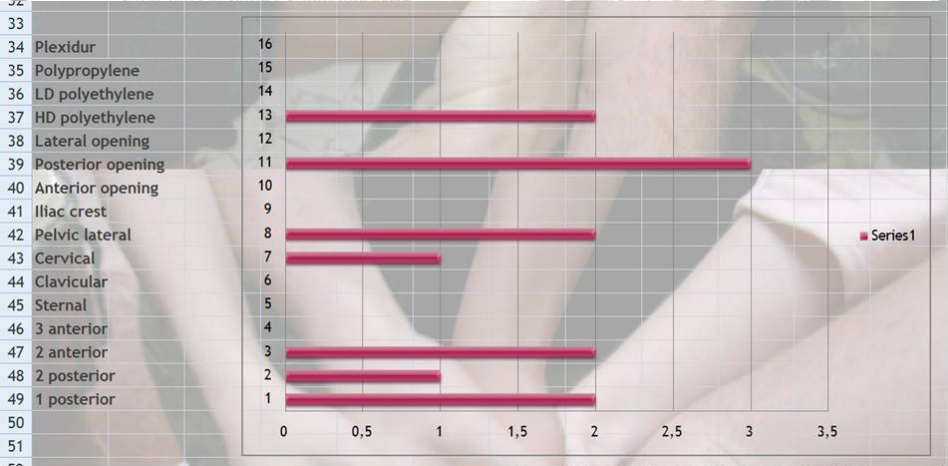
Choose your ideal brace for juvenile kyphosis?

Pressure points | 3 anterior with pelvis, inferior thorax, and sternum (0), 2 anterior point with pelvis and sternum (2), 2 posterior with sacrum and apex kyphosis (1), one posterior with apex kyphosis only (2) [See figure 13]

*Discussion: *There is consensus for polyethylene posterior opening brace, corresponding to the Milwaukee brace.

Q 24 Title: What is the best time for initiating bracing with a rigid kyphosis brace (boy)?

**Figure 14:**
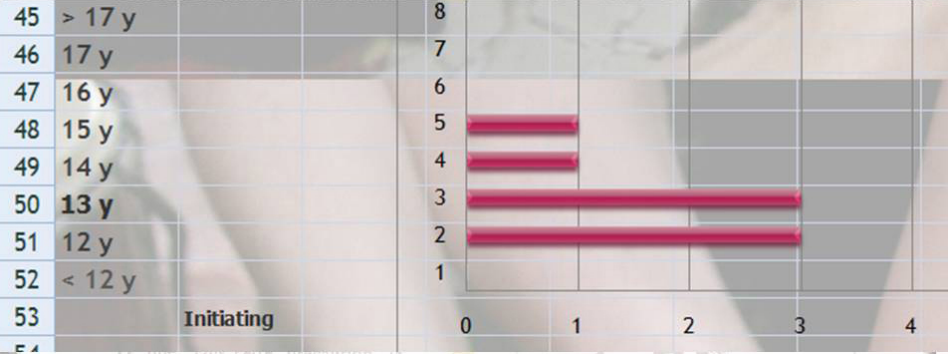
What is the best time for initiating bracing with a rigid kyphosis brace (boy)?

*Results*: < 12 years (0), 12y (3), 13y (3), 14y (1), 15y (1), 16y (0), 17y (0), >17y (0) [See figure 14]

*Discussion*: There is consensus; the best age for bracing seems to be at the beginning of puberty.

*Q25 Title: What is your minimum period to maintain the brace*?

**Figure 15:**
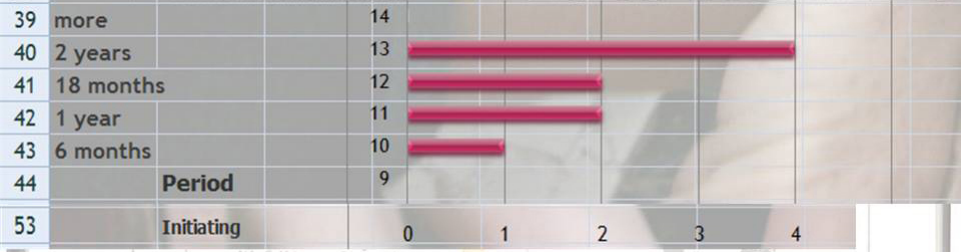
What is your minimum period to maintain the brace?

*Results*: 6 months (1), 1 year (2), 18 months (2), 2 years (4), more (0) [See figure 15]

*Discussion*: Taking into account the age of onset to starting treatment (Q22) there is a consensus to maintain the brace till the end of growth but without waiting for definitive bone maturity at Risser 5.

Q26 Title: What is the best moment for brace weaning?

**Figure 16:**
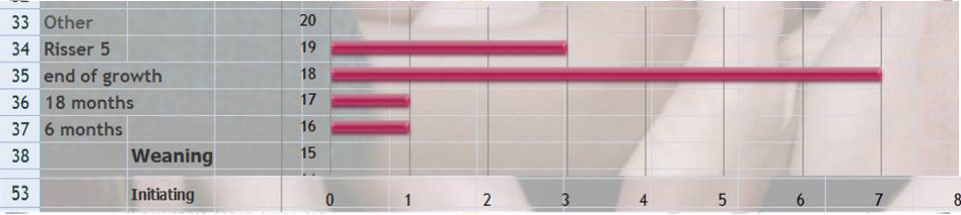
What is the best moment for brace weaning?

*Results*: 6 months (1), 18 months (1), end of growth (7), Risser 5 (3), other (0) [See figure 16]

*Discussion*: This question was asked to verify the consistency of responses. We can confirm that there is a consensus to maintain the brace till the end of growth but without waiting for definitive bone maturity at Risser 5.

## Clinical cases

*Q11 Title: Case N° 1: girl, 13 years, no pain, with idiopathic Kypho-lordotic posture *(figure 5)

**Figure 17:**
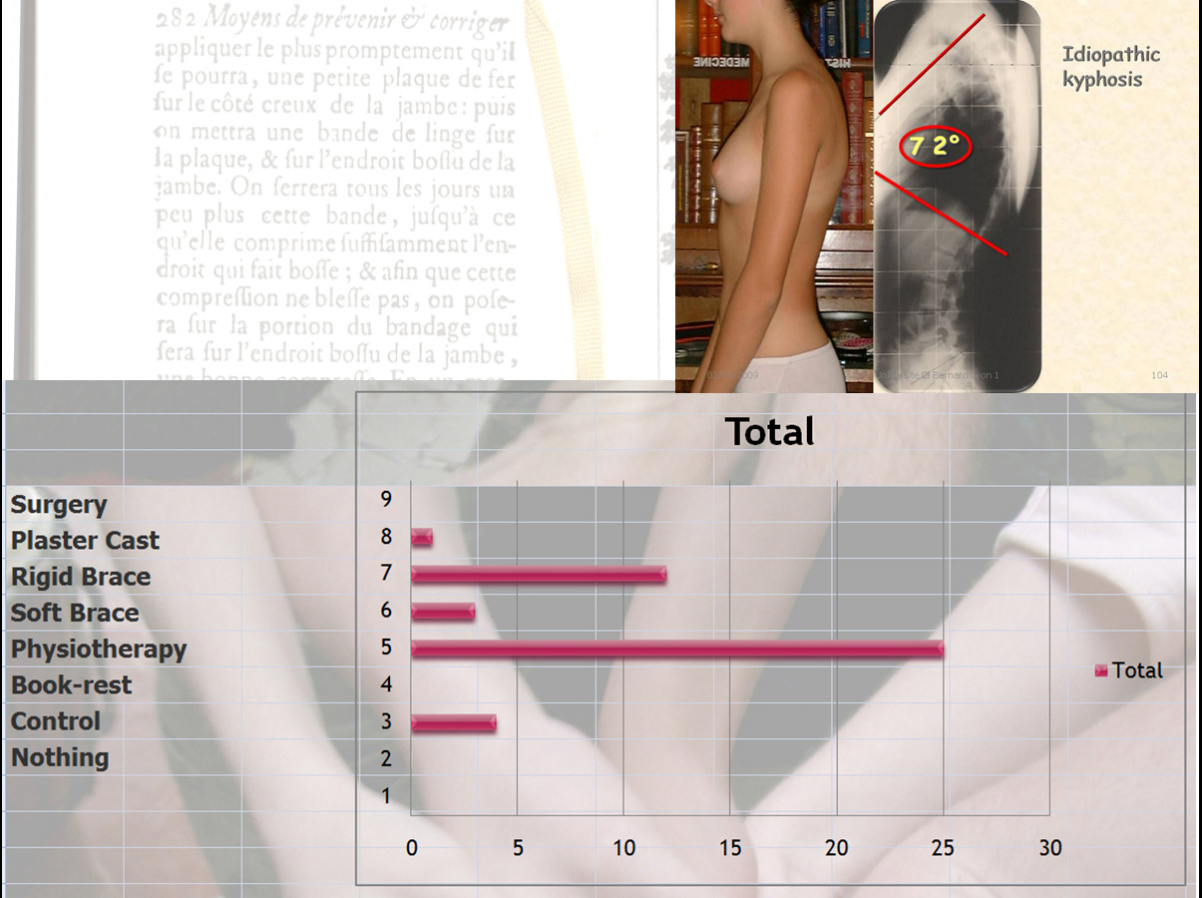
**Case N°1**.

*Results: *Physiotherapy (25/27 = **93%**), Rigid brace (12/27 = **44%**), Control (4/27 = **15%**), Soft brace (3/27 = **11%**), Plaster cast (1/27 = **4%**), Surgery (0 = **0%**), Nothing (0 = **0%**) [See figure 17]

*Discussion: *We have a consensus for physiotherapy and no surgery.

Q12 Title: Case N°2: Boy, 15 years, postural pain, idiopathic, low pelvic incidence

**Figure 18:**
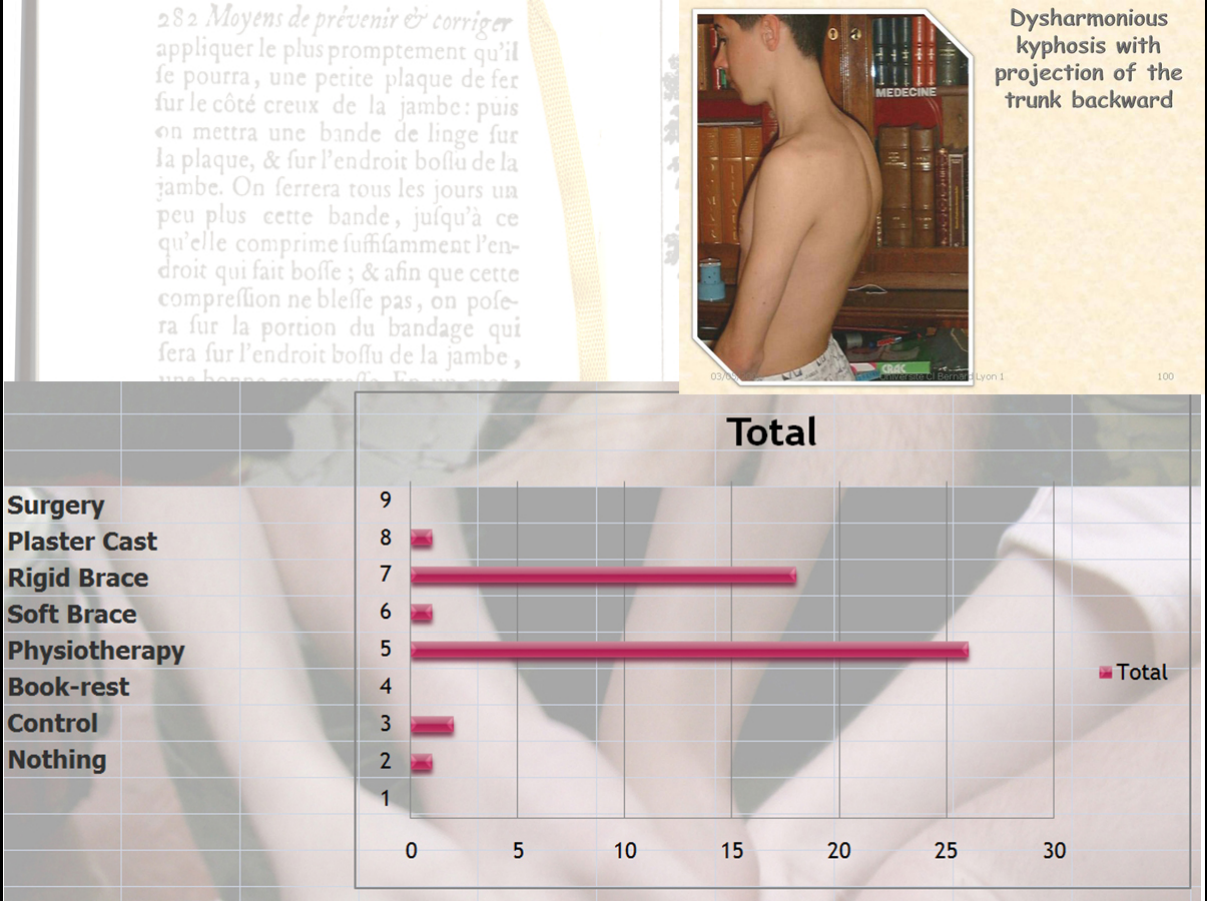
**Case N°2**.

*Results: *Physiotherapy (26/27 = **96%**), Rigid brace (18/27 = **67%**), Control (2/27 = **7%**), Soft brace (1/27 = **4%**), Plaster cast (1/27 = **4%**), Surgery (0 = **0%**), Nothing (0 = **0%**) [See figure 18]

*Discussion: *We have the same consensus for physiotherapy as Q11, but more indications for a rigid brace and consensus to avoid surgery.

Q13 Title: Case N° 3: Boy, 16 years, pain, Scheuermann, Rigid thoracic curve

**Figure 19:**
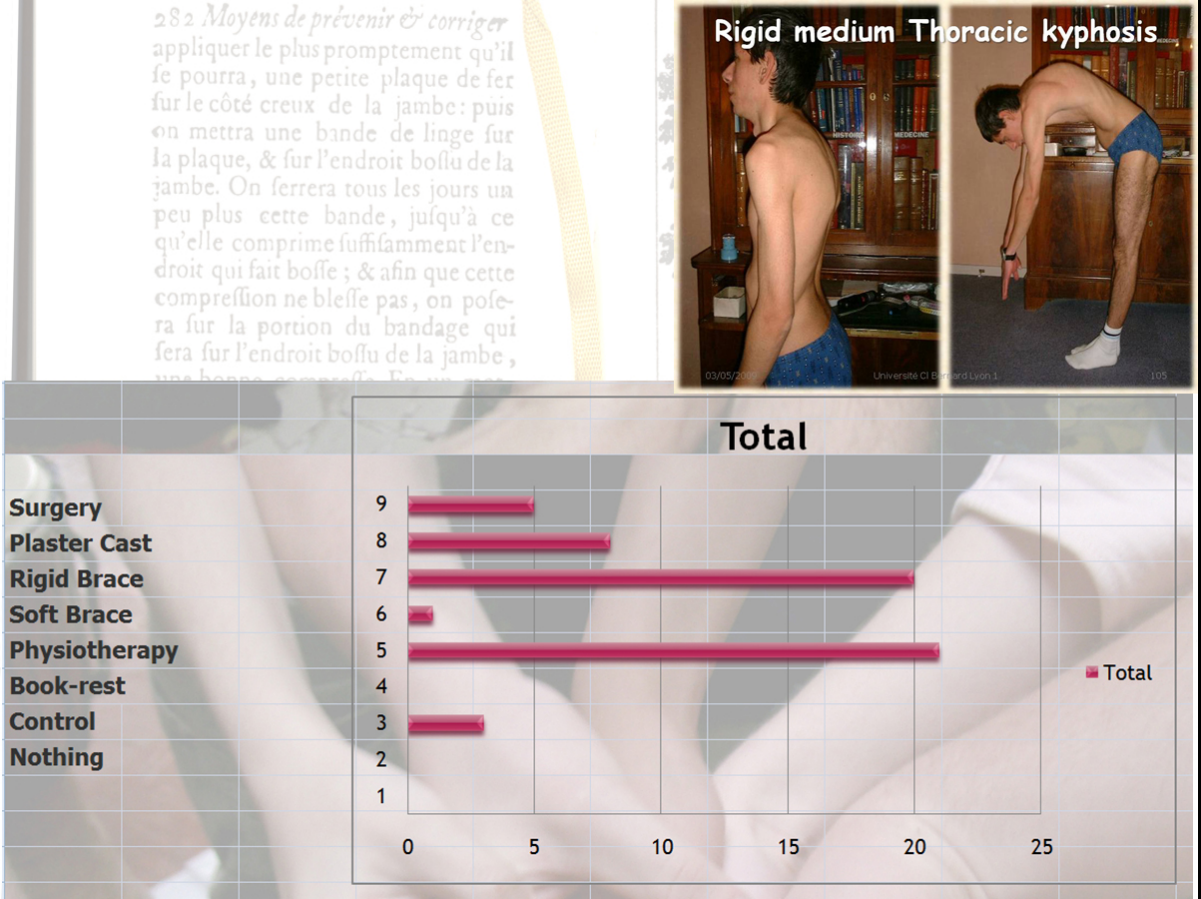
**case N°3**.

*Results: *Physiotherapy (21/27 = **78%**), Rigid brace (20/27 = **74%**), Plaster cast (8/27 = **30%**), Surgery (5/27 = **19%**), Control (3/27 = **11%**), Soft brace (1/27 = **4%**), Nothing (0 = **0%**) [See figure 19]

*Discussion: *We have a consensus for physiotherapy and rigid brace in this case.

Q14 Title: Case N° 4: Girl, 15 years, thoraco-lumbar kyphosis, pain, low pelvic incidence

**Figure 20:**
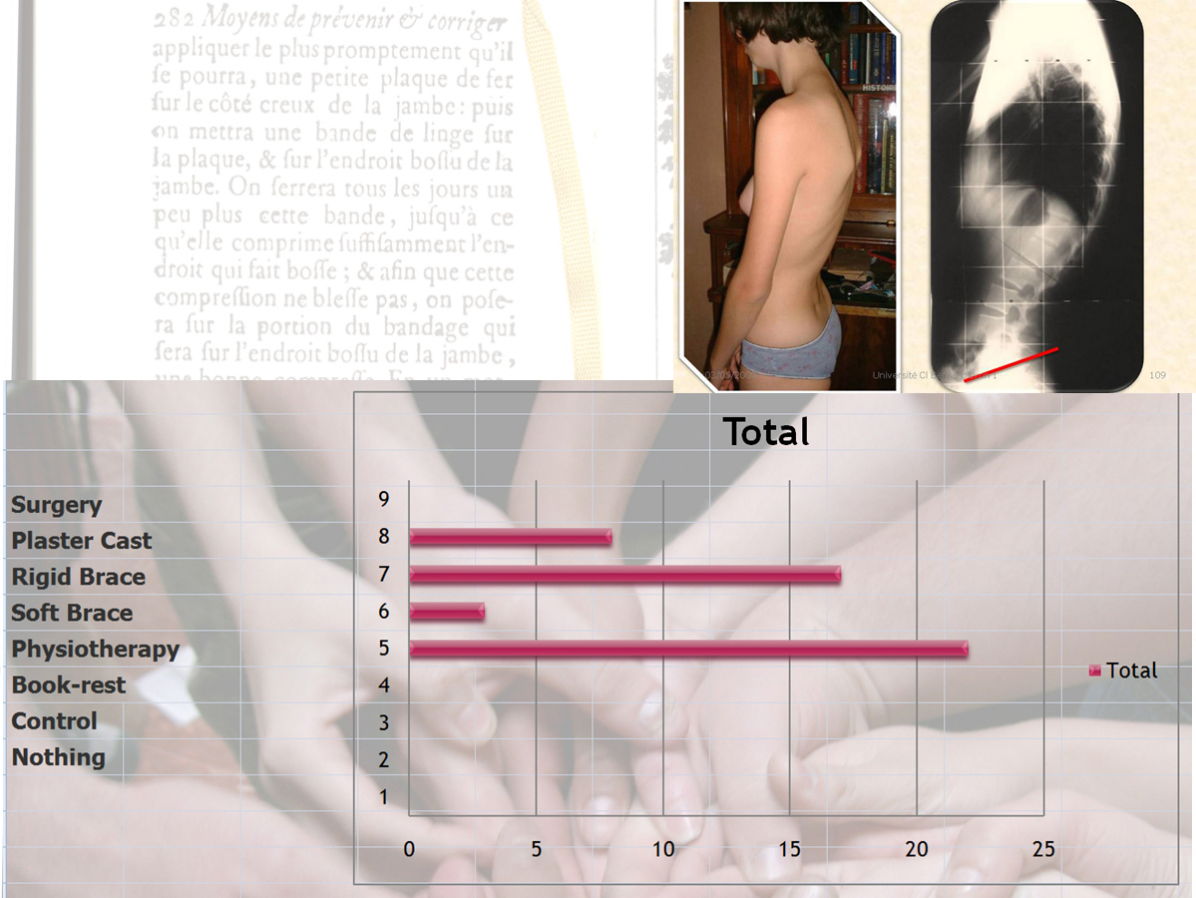
**Case N°4**.

*Results: *Physiotherapy (22/27 = **81%**), Rigid brace (17/27 = **63%**), Plaster cast (8/27 = **30%**), Soft brace (3/27 = **11%**) [See figure 20]

*Discussion: *We have a consensus using physiotherapy and a rigid brace. Unlike the case N°3, there is a consensus against surgery, which emphasizes the importance of conservative treatment for this type of kyphosis.

## Closure questions

Q26 Title: Do you have some suggestions on the questions already prepared?

Results: No (14), Yes (1)

*Comment*: "The treatment of hyperkyphosis should be evaluated according to the aetiology."

*Discussion: Initially, we limited the outset regarding the aetiologies and idiopathic kyphosis. Other causes could require another consensus*.

Q27 Title: Do you want some more questions?

*Results: *No (15), Yes (0)

*Discussion: *We are at the end of the consensus.

## Conclusion: A brief synthesis of the consensus

The experts from SOSORT are convinced of the usefulness of conservative treatment for the management of **Kyphosis **and they practice this treatment daily in their clinical practice. Therefore, is there a need for consensus in the treatment of kyphosis.

The main rehabilitation techniques used are: self postural control and self-elongation. Back school does not seem useful. These physiotherapy exercises should be repeated at home daily for 20 minutes. It is useful before bracing.

The main indications are Scheuermann and pain especially if the kyphosis is rigid.

The biomechanical base for conservative treatment is to decrease mechanical stress on the anterior wall of the vertebral body.

The main indications for early treatment are: rigidity, size of the curve and the Cobb angle.

The best time is at the onset of puberty. The brace should be worn for about 2 years and removed at the end of growth without skeletal maturity at Risser 5.

For a Thoracic Kyphosis

The brace must be worn all night and for part of the day. The most ideal brace is a 4 point system or a 5 point system in case of muscular imbalance.

For a Thoraco-lumbar kyphosis

The brace must be worn during the day in the sitting position and the ideal brace is a 4 point system.

For a juvenile kyphosis

The brace must be worn part time, and the ideal brace is the Milwaukee.

The four clinical cases:

- physiotherapy for muscular idiopathic kypho-lordosis without rigidity.

- brace for an idiopathic painful kyphosis

- Rigid brace and plaster cast for a rigid thoracic or thoraco-lombar dystrophic curve

## Competing interests

The authors declare that they have no competing interests.

## Consent

Written informed consent was obtained from the patients for publication of this report and accompanying images. A copy of the written consent is available for review by the Editor-in-Chief of this journal.

## Authors' contributions

JCdM created the initial and successive questionnaires, chaired this consensus paper at the Lyon SOSORT Meeting, May 23--25, 2009, processed the collected data on a web form, collected the literature and contributed in drafting the manuscript. All co-authors contributed in some way to the improvement of the initial questionnaire and in drafting the manuscript. The other members of the SOSORT board contributed by reviewing, text editing and adding certain text files and references. All authors have read and approved the final manuscript.
